# Supplementary material for: Association between Estimated Cardiorespiratory Fitness and Abnormal Glucose Risk: A Cohort Study
Source: J Clin Med. 2023 Apr 6;12(7):2740. doi: 10.3390/jcm12072740 (PMC10095416; doi:10.3390/jcm12072740)
Supplement: Supplementary file 1 [file jcm-12-02740-s001.zip › jcm-2122494-supplementary.pdf]

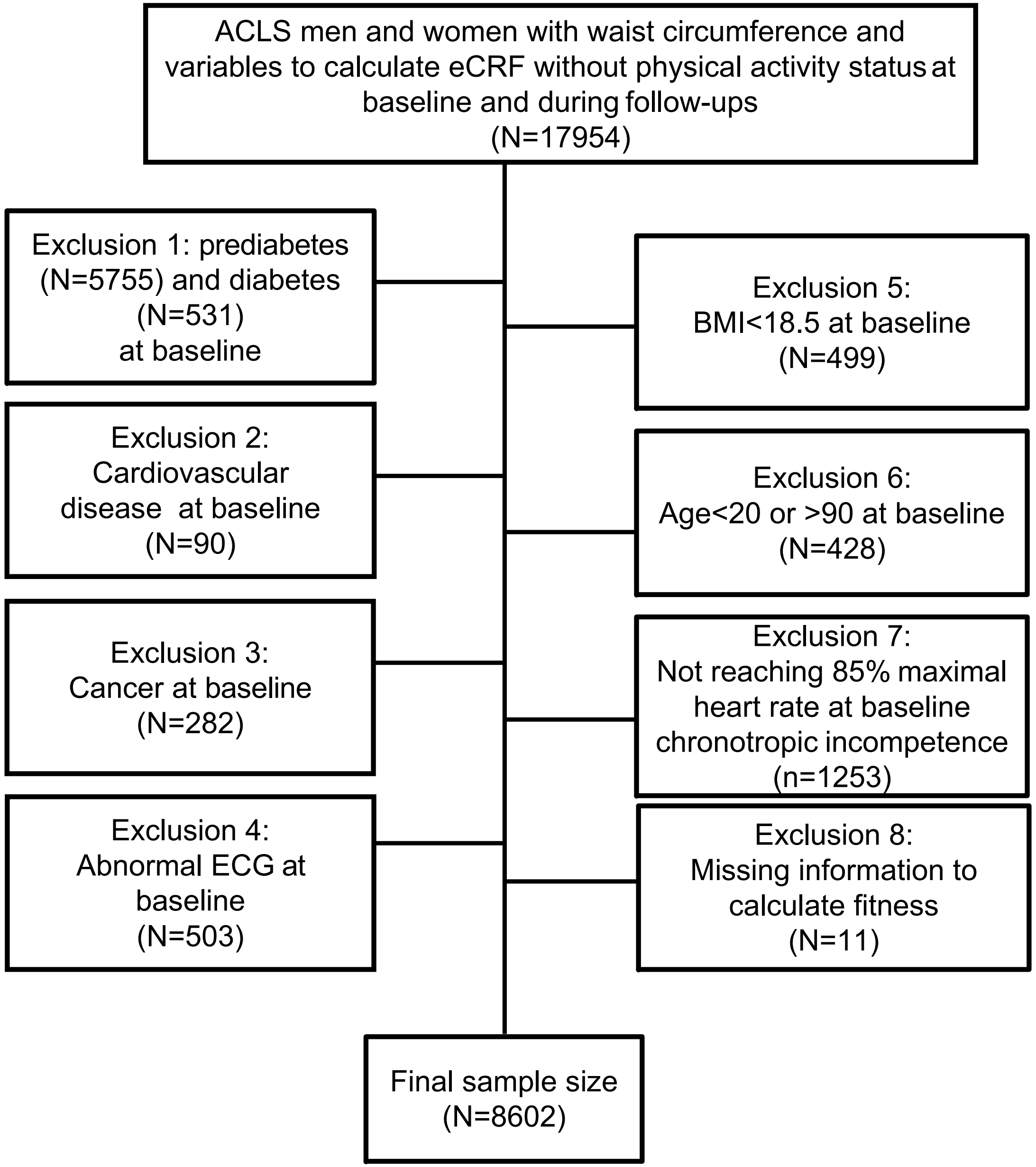

Supplement S1

Figure S1. Study flow and Aerobics Center Longitudinal Study (ACLS) inclusion/exclusion criteria depicting final sample size. BMI, body mass index.

Supplement S2

Men's algorithm

$$
\begin{aligned}
\text{METs} = {} & -0.70\left(\frac{Age-44.14}{9.49}\right)+0.04\left(\frac{Ht-179.15}{6.51}\right)-0.85\left(\frac{rHR-60.10}{10.45}\right)-0.96\left(\frac{BMI-26.46}{3.50}\right)+0.24\left(\frac{SBP-120.58}{12.70}\right)-0.08\left(\frac{DBP-81.09}{9.32}\right)\\
& -0.14\left(\frac{Age-44.14}{9.49}\right)^2-0.05\left(\frac{Age-44.14}{9.49}\right)\left(\frac{Ht-179.15}{6.51}\right)+0.02\left(\frac{Age-44.14}{9.49}\right)\left(\frac{rHR-60.10}{10.45}\right)\\
& +0.05\left(\frac{Age-44.14}{9.49}\right)\left(\frac{BMI-26.46}{3.50}\right)-0.09\left(\frac{Age-44.14}{9.49}\right)\left(\frac{SBP-120.58}{12.70}\right)+0.04\left(\frac{Age-44.14}{9.49}\right)\left(\frac{DBP-81.09}{9.32}\right)\\
& +0.07\left(\frac{Wt-85.04}{12.84}\right)\left(\frac{Ht-179.15}{6.51}\right)-0.07\left(\frac{Ht-179.15}{6.51}\right)^2+0.04\left(\frac{Ht-179.15}{6.51}\right)\left(\frac{rHR-60.10}{10.45}\right)\\
& -0.05\left(\frac{Ht-179.15}{6.51}\right)\left(\frac{BMI-26.46}{3.50}\right)-0.01\left(\frac{Ht-179.15}{6.51}\right)\left(\frac{SBP-120.58}{12.70}\right)+0.11\left(\frac{rHR-60.10}{10.45}\right)^2\\
& +0.13\left(\frac{rHR-60.10}{10.45}\right)\left(\frac{BMI-26.46}{3.50}\right)+0.03\left(\frac{rHR-60.10}{10.45}\right)\left(\frac{SBP-120.58}{12.70}\right)-0.01\left(\frac{rHR-60.10}{10.45}\right)\left(\frac{DBP-81.09}{9.32}\right)\\
& -0.09\left(\frac{BMI-26.46}{3.50}\right)\left(\frac{SBP-120.58}{12.70}\right)+0.04\left(\frac{BMI-26.46}{3.50}\right)\left(\frac{DBP-81.09}{9.32}\right)+0.02\left(\frac{SBP-120.58}{12.70}\right)^2\\
& -0.06\left(\frac{SBP-120.58}{12.70}\right)\left(\frac{DBP-81.09}{9.32}\right)+0.01\left(\frac{DBP-81.09}{9.32}\right)^2+0.88(1-Smoking)+11.29
\end{aligned}
$$

Women's algorithm

$$
\begin{aligned}
METs = {} & -0.71\left(\frac{Age-44.11}{10.17}\right)+0.09\left(\frac{Ht-164.62}{5.96}\right)-0.63\left(\frac{rHR-64.02}{10.11}\right)-0.75\left(\frac{BMI-23.31}{3.68}\right)+0.17\left(\frac{SBP-112.42}{13.94}\right)-0.10\left(\frac{Age-44.11}{10.17}\right)^2\\
& -0.07\left(\frac{Age-44.11}{10.17}\right)\left(\frac{Ht-164.62}{5.96}\right)+0.08\left(\frac{Age-44.11}{10.17}\right)\left(\frac{rHR-64.02}{10.11}\right)+0.10\left(\frac{Age-44.11}{10.17}\right)\left(\frac{BMI-23.31}{3.68}\right)\\
& -0.05\left(\frac{Age-44.11}{10.17}\right)\left(\frac{SBP-112.42}{13.94}\right)+0.02\left(\frac{Wt-63.21}{10.78}\right)\left(\frac{rHR-64.02}{10.11}\right)+0.02\left(\frac{Ht-164.62}{5.96}\right)^2\\
& -0.01\left(\frac{Ht-164.62}{5.96}\right)\left(\frac{BMI-23.31}{3.68}\right)+0.07\left(\frac{Ht-164.62}{5.96}\right)\left(\frac{SBP-112.42}{13.94}\right)+0.10\left(\frac{rHR-64.02}{10.11}\right)^2\\
& +0.05\left(\frac{rHR-64.02}{10.11}\right)\left(\frac{BMI-23.31}{3.68}\right)-0.02\left(\frac{rHR-64.02}{10.11}\right)\left(\frac{DBP-76.04}{9.33}\right)+0.01\left(\frac{BMI-23.31}{3.68}\right)^2\\
& -0.06\left(\frac{BMI-23.31}{3.68}\right)\left(\frac{DBP-76.04}{9.33}\right)-0.03\left(\frac{SBP-112.42}{13.94}\right)\left(\frac{DBP-76.04}{9.33}\right)-0.01\left(\frac{DBP-76.04}{9.33}\right)^2+0.77(1-Smoking)+9.07
\end{aligned}
$$

Google sheet link:

https://docs.google.com/spreadsheets/d/1lnYl4u62AMSUxkzyzKsKBHe8vr3WzLVRYxGyYE7xoGQ/edit#gid=155 8519083
